# Supplementary material for: Plasmon-assisted high-harmonic generation in graphene
Source: Nat Commun. 2017 Feb 22;8:14380. doi: 10.1038/ncomms14380 (PMC5322527; doi:10.1038/ncomms14380)
Supplement: Supplementary Information — Supplementary Figures [file ncomms14380-s1.pdf]

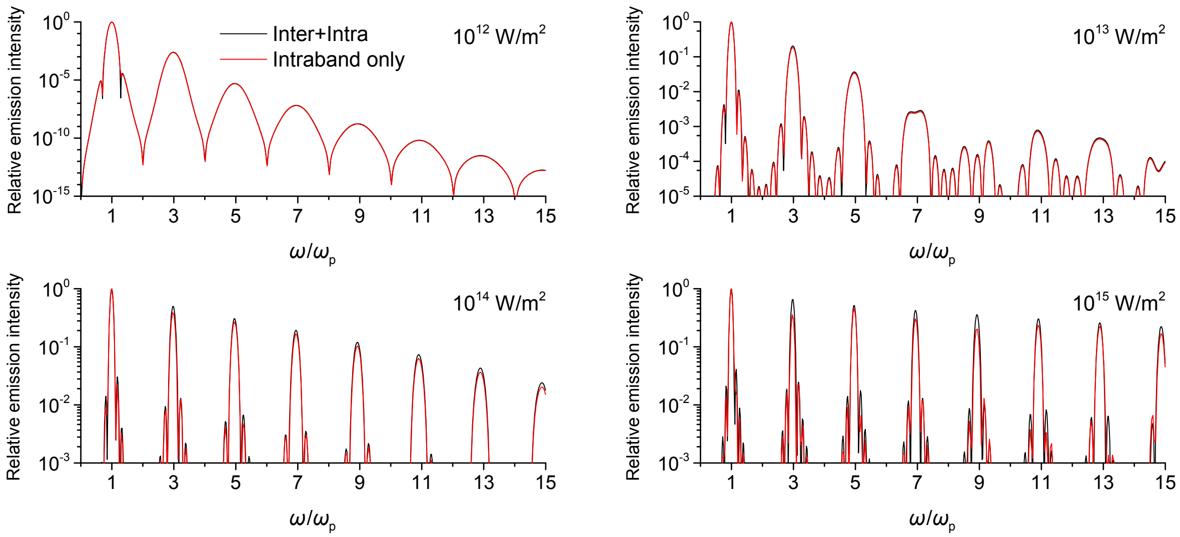

Supplementary Figure 1: Role of interband transitions in plasmon-assisted HHG. We present the spectral decomposition of the light emission energy from a 100 nm-wide graphene ribbon doped to $E_F = 0.4$ eV when illuminated by a normally-incident pulse of 100 fs FWHM duration and centered at the nanoribbon plasmon frequency $\hbar\omega_p = 0.158$ eV. In each panel we consider a peak pulse intensity as indicated in the upper-right corner, and we compare results obtained from MDF-CEM simulations that incorporate both interband and intraband effects to the optical response (black curves) with those including only the intraband contribution (red curves). Interband transitions act to suppress harmonic generation overall, and become more important at higher intensities, but for the parameters considered here (i.e., in the graphene plasmonic regime where $\hbar\omega < 2E_F$), interband effects only marginally suppress harmonic generation.

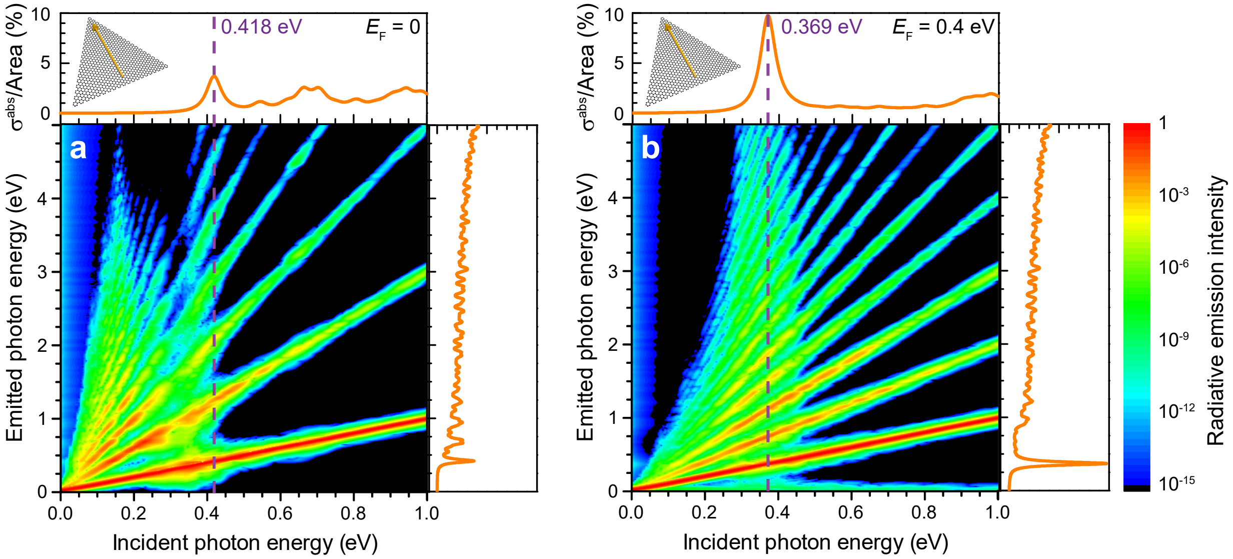

Supplementary Figure 2: Extended HHG data for nanotriangles. We show the same data as in Fig. 4 of the main paper, but with values along each vertical line in the contour plots (i.e., fixed incident photon energy) normalized to the maximum value along that line. For comparison, the upper and right panels alongside each contour show the linear absorption cross-section. In (a), the various features in the linear absorption spectrum of the undoped nanotriangle do not correspond to plasmonic resonances, but rather to discrete electron-hole transitions in the electronic spectrum of the island, and thus do not produce the strong near-field enhancement required for efficient HHG. Relatively strong harmonic generation (but weaker than that of the doped nanotriangle near the plasmon resonance) is then appearing at lower frequencies due to the interplay between (1) an increased proximity to the Dirac point and (2) the overlap of the generated harmonic frequency with a single-electron transition energy.

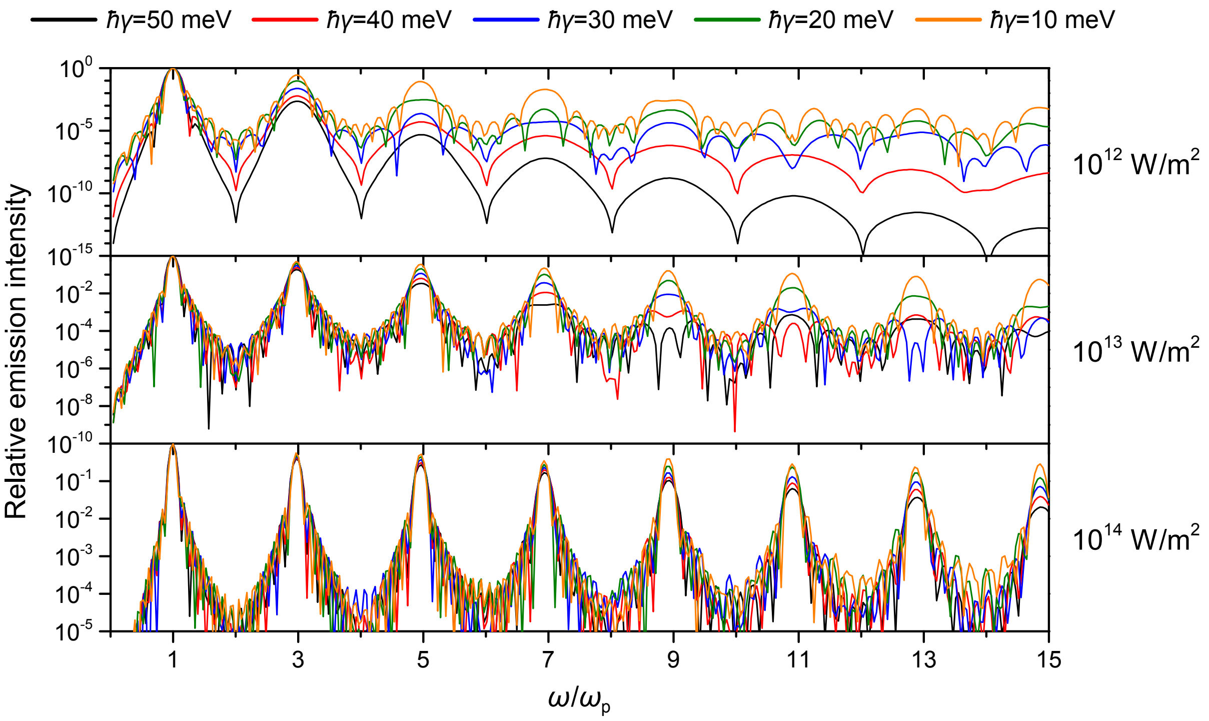

Supplementary Figure 3: **Effect of the relaxation rate.** Based on MDF-CEM simulations, we present the spectral decomposition of the light emission intensity from a 100 nm wide graphene ribbon doped to $E_{\rm F} = 0.4\,$eV when illuminated by a normally-incident pulse of 100 fs FWHM duration and centered at the nanoribbon plasmon frequency $\hbar\omega_{\rm p} = 0.158\,$eV. The three panels correspond to different peak pulse intensities (see labels). The upper legend indicates the values considered for the intrinsic inelastic scattering rate γ. We note that the results presented in the main paper are obtained for a conservative value of the electron scattering lifetime $\tau = 13.2\,$fs, corresponding to the highest scattering rate $\hbar\gamma = 50\,$meV considered here. Attainable graphene samples of longer lifetimes are predicted to improve HHG efficiencies.
